# Change in depressive symptom scores to assess the risk of new-onset dual sensory impairment in middle-aged and older adults: a nationwide cohort study

**DOI:** 10.3389/fpubh.2025.1520552

**Published:** 2025-03-26

**Authors:** Jiang Wang, Lanzhi Duan, Rui Zeng, Fayi Xie, Zhigang Wu, Yanhong Luo, Xiaoming Zhang, Wei Li, Dongmei Ye, Ke Zhu, Tenghui Fan, Aizhang Zhu, Lihuan Chen, Wan Zhu, Yueyue Zhao, Hongmei Hu, Yi Xiao

**Affiliations:** ^1^School of Basic Medicine, Health Science Center, Jinggangshan University, Ji'an, Jiangxi, China; ^2^Online Collaborative Research Center for Evidence-Based Medicine Ministry of Education, JingGangshan Univesity Branch, Ji'an, Jiangxi, China; ^3^School of Clinical Medicine, Health Science Center, Jinggangshan University, Ji'an, Jiangxi, China; ^4^Department of Emergency, The People's Hospital of Baoan Shenzhen, Shenzhen, China; ^5^Affiliated Stomatological Hospital of Jinggangshan University, Center for Clinical Medicine Research of Jinggangshan University, Ji'an, China; ^6^School of Chinese Medicine, Jinggangshan University, Ji'an, Jiangxi, China; ^7^School of Mathematics and Physics, Jinggangshan University, Ji'an, Jiangxi, China

**Keywords:** CHARLS, depressive symptom, DSI, middle-aged and older adult, K-means clustering

## Abstract

**Background:**

Evidence of the association between change in depressive symptom scores and new-onset dual sensory impairment (DSI) remain underexplored. We aimed to investigate the relationship between depressive symptom scores and the risk of new-onset DSI in middle-aged and older adults.

**Methods:**

The study included 3,237 participants. The Center for Epidemiologic Studies Depression Scale (CES-D-10) was used to assess depression symptoms. CES-D-10 scores were classified according to the K-means cluster analysis. Subsequently, the logistic regression analysis was used to determine the association between the change in CES-D-10 scores and DSI. Additionally, restricted cubic splines (RCS) were employed to examine whether there was a linear correlation between cumulative CES-D-10 scores and DSI. We also explored whether the association of CES-D-10 scores with DSI varies across different subgroups.

**Results:**

One thousand and sixty one out of 3,237 participants had a new-onset DSI during 3 years. Compared to class 1 with the best control of depressive state, the OR for class 2 with good control was 1.54 (95% CI, 1.28, 1.85), the OR for class 3 with moderate control was 1.59 (95% CI, 1.22, 2.07), the OR for class 4 with worse control was 1.62 (95% CI, 1.31, 2.02), and the OR for class 5 with consistently high levels was 1.86 (95% CI, 1.34, 2.57).

**Conclusions:**

Individuals exhibiting suboptimal management of depressive symptoms were more susceptible to developing new-onset DSI, underscoring the need for early depression screening and assessment in the preventative strategies for new-onset DSI.

## 1 Introduction

Sensory impairments (SIs), including hearing disturbances (HI), visual disturbances (VI), and dual sensory disturbances (DSI). SIs are highly prevalent in older adults and increase with age (1). The World Health Organization (WHO) reported that at least 2.2 billion people suffer from some degree of VI or blindness. And worldwide, nearly 2.5 billion people are expected to suffer from some degree of HI by 2050, causing a significant burden on achieving public health goal (2, 3). Previous studies have shown that SIs was associated with mental health, poor quality of life, increased risk of dementia, physical function and even mortality (4–9). Researchers have found that HI and VI were positively associated with mortality, while DSI worsens the effect of a single SI (10, 11). Moreover, the prevalence of DSI in the older adult in China is 58.6%. This incidence far exceeds that of other developed or developing countries (12).

Depression is a common mental disorder that affects about 350 million people worldwide, according to the WHO (13). Depression can lead to loss of interest in life, insomnia, mood abnormalities, and pose a threat to the physical and mental health of individuals. In addition, major depression leads to greatly increased suicidal thinking and behavior (14). It is estimated that more than one million people worldwide complete suicide due to depression each year (15). In recent years, the situation of middle-aged and older adult people suffering from depression in China is not optimistic. Depression is prevalent in the middle-aged and older adult Chinese population at a rate of 17.4% to 46.15%, according to previous studies (16).

Emerging epidemiological studies suggest that treating or rehabilitating hearing or vision disorders will help prevent depression (17). Another study suggests that interventions targeting only modifiable VI and DSI can prevent depressive symptoms (18). However, few studies have investigated the changes in depression scores and the incidence of DSI, and results from previous studies suggest a bidirectional association between vision loss and depression in older adults (19, 20). Another meta-analysis, which included 20 studies and 675,291 individuals, demonstrated a significant bidirectional association between sensorineural hearing loss (SNL) and depression. And these associations were further influenced by age, region, and diagnostic methods (21). A study based on the China Health and Retirement Longitudinal Study (CHARLS) database suggests a bidirectional association between SI and baseline depression (22). Previous studies have only considered the predictive effect of baseline depression status on SI and have yielded inconsistent results due to different definitions of depression on different scales.

Therefore, it is unclear whether changes in depression scores in older adults have prognostic value for new-onset DSI in middle-aged and older adults. This study aimed to evaluate whether the level and the change in depressive symptom scores can predict the incidence of DSI in people aged 45 years and older using data from the CHARLS.

## 2 Methods

### 2.1 Study population

This prospective cohort studies are based on data from the CHARLS database from 2013, 2015, and 2018. CHARLS is a nationally representative longitudinal survey of Chinese as well as spouses aged 45 years or older to analyze the aging of the Chinese population (23). The baseline national wave of CHARLS is being fielded in 2011. The baseline survey used a multistage probability sampling approach covering 150 county-level, 450 village level, and about 17,000 people in 10,000 households. These samples were followed every 2 to 3 years from 2011–2012 (wave 1). Since then, the national follow-up survey has been continued in 2013, 2015, 2018, 2020, and 2021–23. CHARLS has published a total of four waves of follow-up data so far (wave 2 in 2013, wave 3 in 2015, wave 4 in 2018, and wave 5 in 2020).

**Figure 1:**
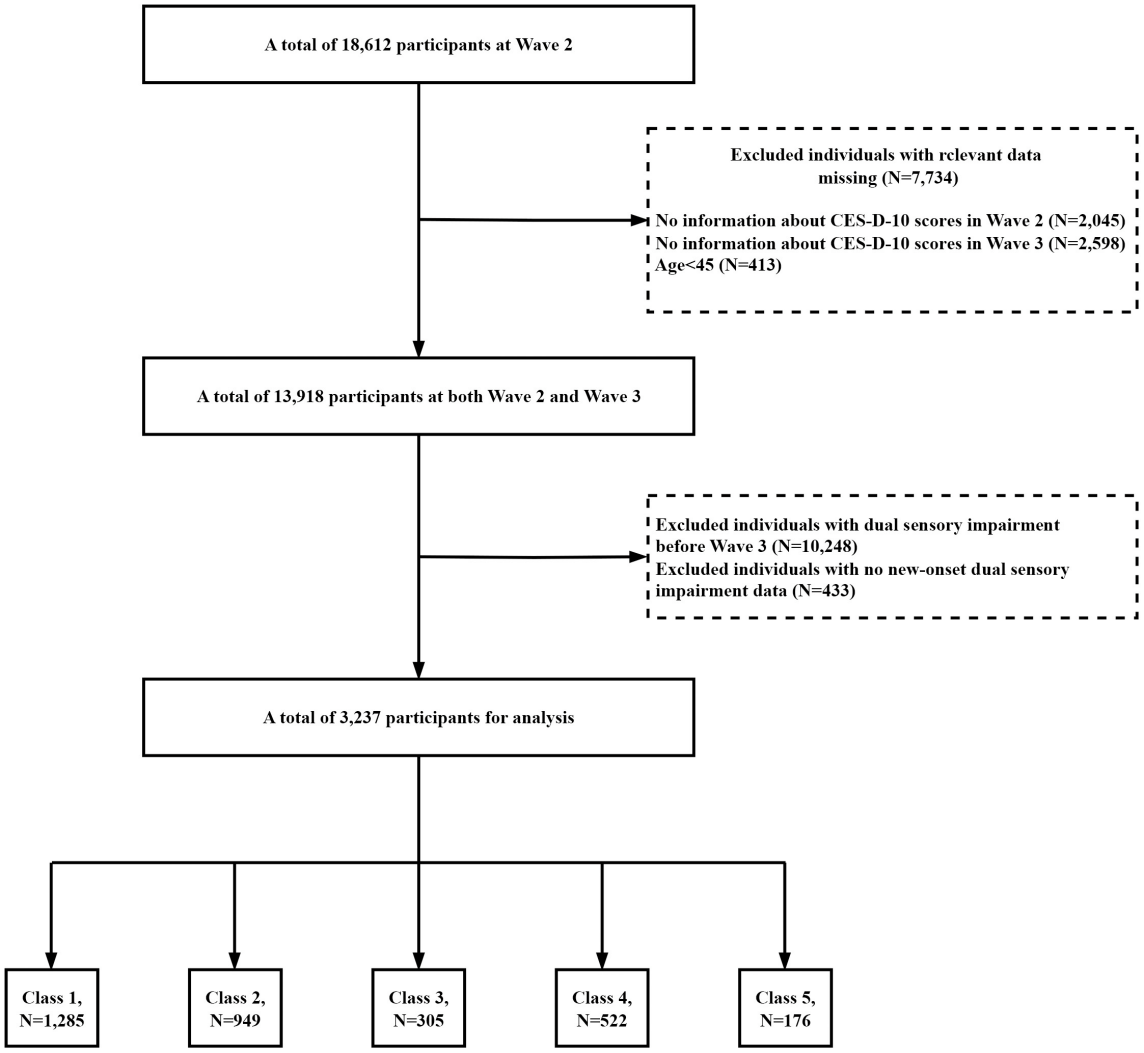
Flowchart of the study population.

In our study, a total of 18,612 participants were initially surveyed at Wave 2. We included participants who had to have data on depression scores from Wave 2 and Wave 3 and who were 45 years of age or older. In addition, we included participants who did not have an outcome event prior to Wave 3 and had Wave 4 outcome event data. Figure 1 shows the selection process for the study population. Of the 18,612 participants in CHARLS, we first excluded 2,045 participants with missing the 10-item Center for Epidemiologic Studies Depression Scale (CESD-10) scores data at baseline, and secondly, we excluded 2,598 participants with missing CESD-10 scores data at Wave 3. In addition, 10,248 participants with DSI before 2015 or loss to follow-up were also excluded. Eventually 3,237 eligible participants were included in the analysis. The Biomedical Ethics Review Board of Peking University approved the ethical aspects of the CHARLS data collection process (IRB00001052-11015), and informed consent was obtained from all participants.

### 2.2 Assessment of depressive symptoms

Our study assessed depression among respondents using CESD-10 (24, 25). The CESD-10 was used to measure depressive symptoms in the CHARLS questionnaire. CES-D-10 is highly reliable and valid for successful measurement of depressive symptoms in middle-aged and older adults (26). Previous studies have shown reasonable sensitivity (0.85) and specificity (0.80) of CES-D-10 in Chinese adults. CESD-10 has satisfactory reliability in terms of internal consistency (Cronbach's alpha = 0.78–0.79) (27). The scale primarily pertains to the psychological situation of the interviewees in the previous week. It covers ten questions: “I was bothered by things that do not usually bother me,” “I had trouble keeping my mind on what I was doing,” “I felt depressed,” “I felt everything I did was an effort,” “I felt hopeful about the future,” “I felt fearful,” “My sleep was restless,” “I was happy,” “I felt lonely,” and “I could not get going.” The answers on the CES-D-10 were scored on a 4-level scale: 0 = little or no (<1day), 1 = not much (1–2 days), 2 = sometimes or half of the time (3–4 days), and 3 = most of the time (5–7 days). A total of ten scores were added together to determine the final score. The respondents' depressive scores range between 0 and 30. Depression symptoms are identified in individuals with scores >10 (15, 28). A higher score indicates a higher level of depression, and vice versa.

The exposure variables used in this study were measures of depressive symptom scores in 2013 and 2015. The cumulative depressive symptom scores was determined by the expression: (CES-D scores2013+CES-D scores2015)/2^*^time [2015–2013] (29). More information about the CHARLS could be obtained at: http://charls.pku.edu.cn/.

### 2.3 Dual sensory impairment

In the CHARLS questionnaire, two questions are used to assess VI: (1) How good is your vision for seeing things at a distance (with glasses or corrective lenses), like recognizing a friend from across the street? (2) How good is your vision for seeing things up close (with glasses or corrective lenses), like reading ordinary newspaper print?

For each question, the answer options include “excellent,” “very good,” “good,” “fair,” or “poor.” We found that respondents reporting “fair” or “poor” (for either long distance or near vision) were classified as having VI.

Regarding the assessment of HI, the question is: Is your hearing excellent, very good, good, fair, or poor (with a hearing aid if you normally use it and without if you normally don't)? If participants report “fair,” or “poor hearing,” they are considered to have HI. DSI was considered to exist when HI and VI were both present. Such assessment of SI has been widely used in previous CHARLS related studies (30–32).

### 2.4 Covariates

Demographic Characteristics, lifestyle factors, and health-related factors were considered as potential confounding variables. Demographic characteristics included age, gender, hukou, educational level, and marital status. In China, Hukou is divided into agricultural and non-agricultural hukou (villagers and urban residents) based on their rural or urban hukou identity inherited from their parents at birth (33). Lifestyle factors included smoking status and alcohol drinking. Health-related factors included history of hypertension, diabetes, heart disease, stroke, dyslipidemia, and lung disease (34).

### 2.5 Statistical analysis

There are some data missing in the CHARLS database, and the missing number and proportion of each variable are shown in Supplementary Table S1. In order to ensure the accuracy of the research results, Template method (R Package “VIM”) (35) and multiple interpolation method (R Package “mice”) (36) were used to fill them. The predictive mean matching method of predictive mean matching was used and 5 cycles of imputation were performed (m = 5), A density plot based on imputed data was generated using the densityplot function to demonstrate differences between different imputation methods. Adjusted variables included hukou, smoking status, drinking status, hypertension, diabetes, heart disease, stroke, dyslipidemia, and lung disease. Five datasets were averaged to obtain the final imputation data set for the analysis.

**Figure 2:**
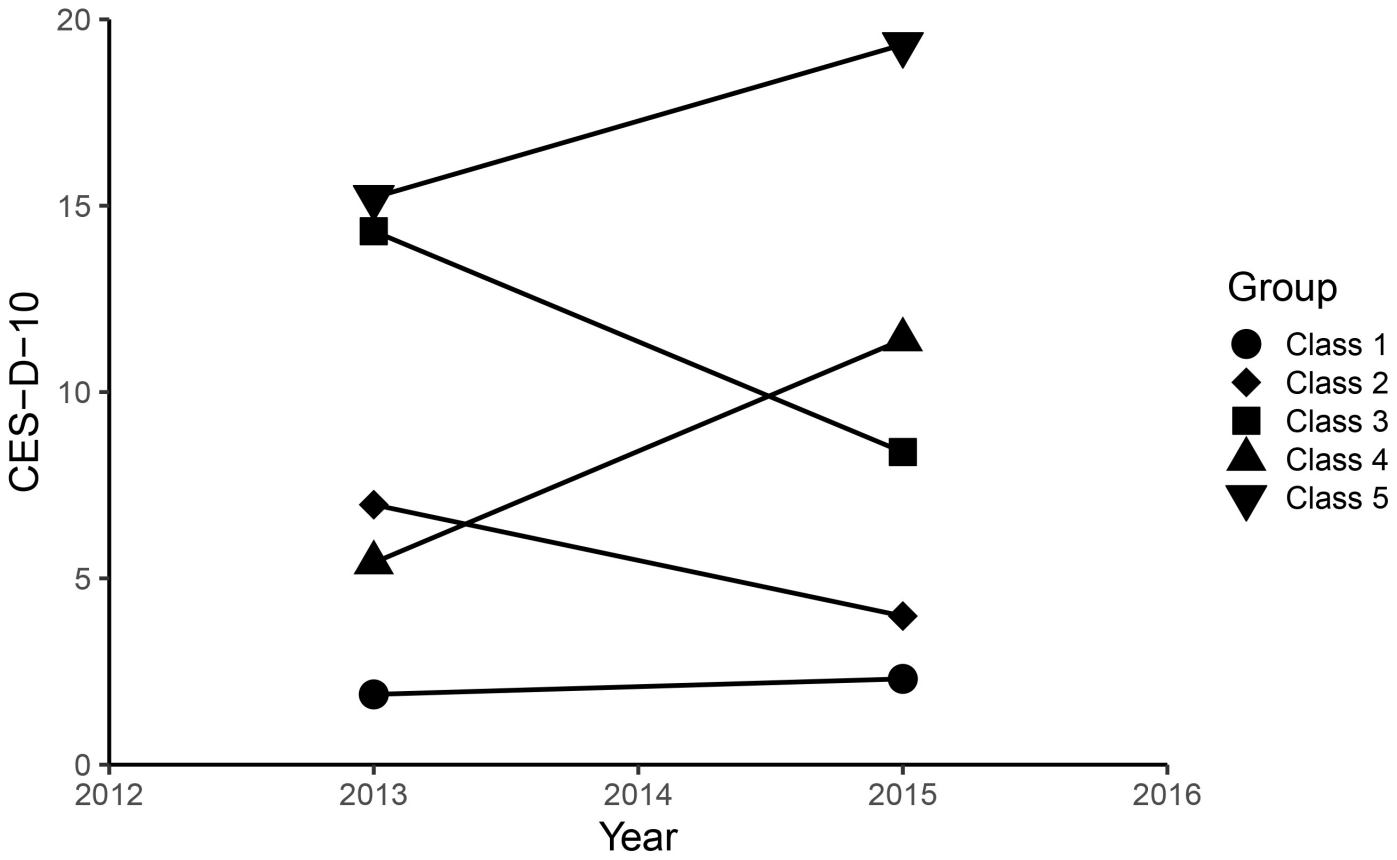
The CES-D-10 scores clustering by k-means clustering.

CES-D-10 scores in 2012 and 2015 were grouped using an unsupervised machine learning technique called K-means with Euclidean distance. Each observation is assigned to the cluster with the closest mean value, which serves as the prototype of the cluster. K-means cluster analysis was used to classify the CES-D-10 transition data into 5 classes (Figure 1). The results of clustering for each classes were as follows: for class 1 (*n* = 1,285), the CES-D-10 ranged from 1.89 ± 1.51 to 2.30 ± 2.18, and the mean (SD) cumulative CES-D-10 was 4.19 ± 2.65, representing best control of CES-D-10; for class 2 (*n* = 949), the CES-D-10 ranged from 6.98 ± 1.89 to 3.99 ± 2.18, and the mean (SD) cumulative CES-D-10 was 10.97 ± 2.66, representing good control of CES-D-10; for class 3 (*n* = 305), the CES-D-10 ranged from 14.31 ± 3.27 to 8.39 ± 3.24, and the mean (SD) cumulative CES-D-10 was 22.70 ± 4.57, representing moderate control of CES-D-10; for class 4 (*n* = 522), the CES-D-10 ranged from 5.42 ± 2.58 to 11.41 ± 3.03, and the mean (SD) cumulative CES-D-10 was 16.83 ± 4.21, representing worse control of CES-D-10; for class 5 (*n* = 176), the CES-D-10 ranged from 15.22 ± 5.03 to 19.32 ± 3.81, and the mean (SD) cumulative CES-D-10 was 34.55 ± 5.68, representing the worst control of CES-D-10 (Figure 2).

Logistic regression was used to investigate the relationship between depression scores and DSI. Three models were estimated: Model I adjusted for age and gender; Model II adjusted for factors in model I and educational level, marital status, hukou, smoking status, drinking status; Model III adjusted for factors in model II and history of hypertension, diabetes, heart disease, stroke, dyslipidemia and lung disease. A restricted cubic spline (RCS) regression model using four nodes (20^th^, 40^th^, 60^th^, and 80^th^) was then used to test whether there was a linear relationship between cumulative CES-D-10 scores and new-onset of DSI. In addition, we performed stratified analyses and calculated the magnitude of interaction between them to exclude the effect of potential confounders on the study results. Results were presented as OR (95%CI) *P*-value. Statistical analyses were completed using R4.1.0 software and EmpowerRCH4.1.

## 3 Results

### 3.1 Study population and baseline characteristics

Demographic and baseline characteristics of the different CES-D-10 categories included in the participants were summarized in Table 1.

**Table 1:** Characteristics of the study according to the change of CES-D-10 Scores.

**Characteristics**	**Total**	**Class 1**	**Class 2**	**Class 3**	**Class 4**	**Class 5**	***P*-value**
*n*	3,237	1,285	949	305	522	176	
Age	57.45 ± 8.73	57.17 ± 8.56	57.46 ± 8.71	57.82 ± 8.93	57.55 ± 8.91	58.43 ± 9.02	0.388
Gender (male)	1,675 (51.75%)	758 (58.99%)	509 (53.64%)	120 (39.34%)	244 (46.74%)	44 (25.00%)	<0.001
**Education level**							<0.001
Primary school or lower	1,849 (57.12%)	627 (48.79%)	532 (56.06%)	208 (68.20%)	341 (65.33%)	141 (80.11%)	
Secondary school or higher	1,388 (42.88%)	658 (51.21%)	417 (43.94%)	97 (31.80%)	181 (34.67%)	35 (19.89%)	
Current married	2,921 (90.24%)	1,202 (93.54%)	855 (90.09%)	255 (83.61%)	466 (89.27%)	143 (81.25%)	<0.001
**Hukou**							<0.001
Agriculture	2,433 (75.16%)	892 (69.42%)	722 (76.08%)	254 (83.28%)	413 (79.12%)	152 (86.36%)	
Others	804 (24.84%)	393 (30.58%)	227 (23.92%)	51 (16.72%)	109 (20.88%)	24 (13.64%)	
Smoking	870 (26.88%)	382 (29.73%)	259 (27.29%)	80 (26.23%)	122 (23.37%)	27 (15.34%)	<0.001
Drinking	1,251 (38.65%)	575 (44.75%)	376 (39.62%)	84 (27.54%)	182 (34.87%)	34 (19.32%)	<0.001
Hypertension	774 (23.91%)	306 (23.81%)	215 (22.66%)	85 (27.87%)	117 (22.41%)	51 (28.98%)	0.160
Diabetes	200 (6.18%)	68 (5.29%)	63 (6.64%)	23 (7.54%)	34 (6.51%)	12 (6.82%)	0.510
Heart disease	312 (9.64%)	110 (8.56%)	76 (8.01%)	41 (13.44%)	46 (8.81%)	39 (22.16%)	<0.001
Stroke	62 (1.92%)	15 (1.17%)	21 (2.21%)	10 (3.28%)	7 (1.34%)	9 (5.11%)	0.001
Dyslipidemia	390 (12.05%)	152 (11.83%)	113 (11.91%)	41 (13.44%)	61 (11.69%)	23 (13.07%)	0.928
Lung disease	234 (7.23%)	79 (6.15%)	66 (6.95%)	26 (8.52%)	42 (8.05%)	21 (11.93%)	0.051
Depressive symptoms	594 (18.35%)	0 (0.00%)	111 (11.70%)	305 (100.00%)	22 (4.21%)	156 (88.64%)	<0.001
CES-D-10_2013_	5.84 ± 4.85	1.89 ± 1.51	6.98 ± 1.89	14.31 ± 3.27	5.42 ± 2.58	15.22 ± 5.03	<0.001
CES-D-10_2015_	5.76 ± 5.29	2.30 ± 2.18	3.99 ± 2.18	8.39 ± 3.24	11.41 ± 3.03	19.32 ± 3.81	<0.001
Cumulative CES-D-10	11.61 ± 8.83	4.19 ± 2.65	10.97 ± 2.66	22.70 ± 4.57	16.83 ± 4.21	34.55 ± 5.68	<0.001
Dual sensory impairment	1,061 (32.78%)	343 (26.69%)	341 (35.93%)	112 (36.72%)	194 (37.16%)	71 (40.34%)	<0.001

Continuous variables were expressed as mean ± standard deviation (SD) in case of normal distribution and compared between two groups by Kruskal-Wallis rank sum test. If the count variable had a theoretical number <10, Fisher's exact probability test was used. Categorical variables are presented as counts (percentages) and compared by Chi-square test.

Three thousand two hundred and thirty seven participants were selected for analysis in this study (of whom 1,675 were male), with an average age of 57.45 ± 8.73. The mean CES-D-10 was 5.84 ± 4.85 and 5.76 ± 5.29, respectively. And the mean (SD) cumulative CES-D-10 was 11.61 ± 8.83. In summary, compared with class 1, participants in class 5 had a higher mean age, with more female and rural hukou, lower proportion of higher education and marriage, and had fewer current smokers and drinkers, a higher prevalence of hypertension, diabete, heart disease, stroke, dyslipidemia, and lung disease.

### 3.2 Association between CES-D-10 scores and new-onset dual sensory impairment

The relationship between different CES-D-10 scores and new-onset DSI is presented in Table 2.

**Table 2:** Logistic regression analysis for the association between different classes and dual sensory impairment.

**Cluster**	**Crude**	**Model I**	**Model II**	**Model III**
	**OR (95%CI)** ***P*****-value**	**OR (95%CI)** ***P*****-value**	**OR (95%CI)** ***P*****-value**	**OR (95%CI)** ***P*****-value**
**Change in the CES-D-10 scores**				
Class 1	Ref	Ref	Ref	Ref
Class 2	1.54 (1.28, 1.85) <0.001	1.53 (1.28, 1.84) <0.001	1.50 (1.25, 1.80) <0.001	1.50 (1.25, 1.80) <0.001
Class 3	1.59 (1.22, 2.07) <0.001	1.57 (1.21, 2.05) <0.001	1.50 (1.15, 1.97) 0.003	1.50 (1.15, 1.97) 0.003
Class 4	1.62 (1.31, 2.02) <0.001	1.61 (1.30, 2.01) <0.001	1.55 (1.24, 1.93) <0.001	1.54 (1.24, 1.92) <0.001
Class 5	1.86 (1.34, 2.57) <0.001	1.82 (1.30, 2.53) <0.001	1.70 (1.21, 2.37) 0.002	1.68 (1.20, 2.35) 0.003
**Cumulative CES-D-10 scores**				
Q1	Ref	Ref	Ref	Ref
Q2	1.48 (1.14, 1.91) 0.003	1.46 (1.13, 1.89) 0.004	1.42 (1.09, 1.84) 0.008	1.42 (1.09, 1.84) 0.008
Q3	1.58 (1.22, 2.05) <0.001	1.56 (1.20, 2.02) <0.001	1.51 (1.16, 1.96) 0.002	1.50 (1.16, 1.95) 0.002
Q4	2.05 (1.59, 2.64) <0.001	2.03 (1.57, 2.61) <0.001	1.93 (1.49, 2.49) <0.001	1.93 (1.50, 2.50) <0.001
Q5	1.98 (1.53, 2.55) <0.001	1.93 (1.49, 2.51) <0.001	1.81 (1.39, 2.35) <0.001	1.80 (1.38, 2.34) <0.001
**Depressive symptoms**				
CES-D-10 ≤ 10	Ref	Ref	Ref	Ref
CES-D-10 > 10	1.31 (1.09, 1.57) 0.005	1.28 (1.06, 1.54) 0.010	1.23 (1.02, 1.49) 0.033	1.23 (1.02, 1.49) 0.033

Crude, adjusted for none. Model I, adjusted for age, gender. Model II, adjusted for age, gender, education, marital status, Hukou, smoking status, and drinking status. Model III, adjusted for factors in model II and history of hypertension, diabetes, heart disease, stroke, dyslipidemia, and lung disease. The CESD scores, measured by the 10-item Center for Epidemiologic Studies Depression Scale, varies between 0 and 30, and then it was split into quartiles. Q1 (<4), Q2 (4, 8), Q3 (8, 12), Q4 (12, 18), Q5 (>18). Defined as a score of 10 or greater on the 10-item center for epidemiologic studies depression scale.

**Figure 3:**
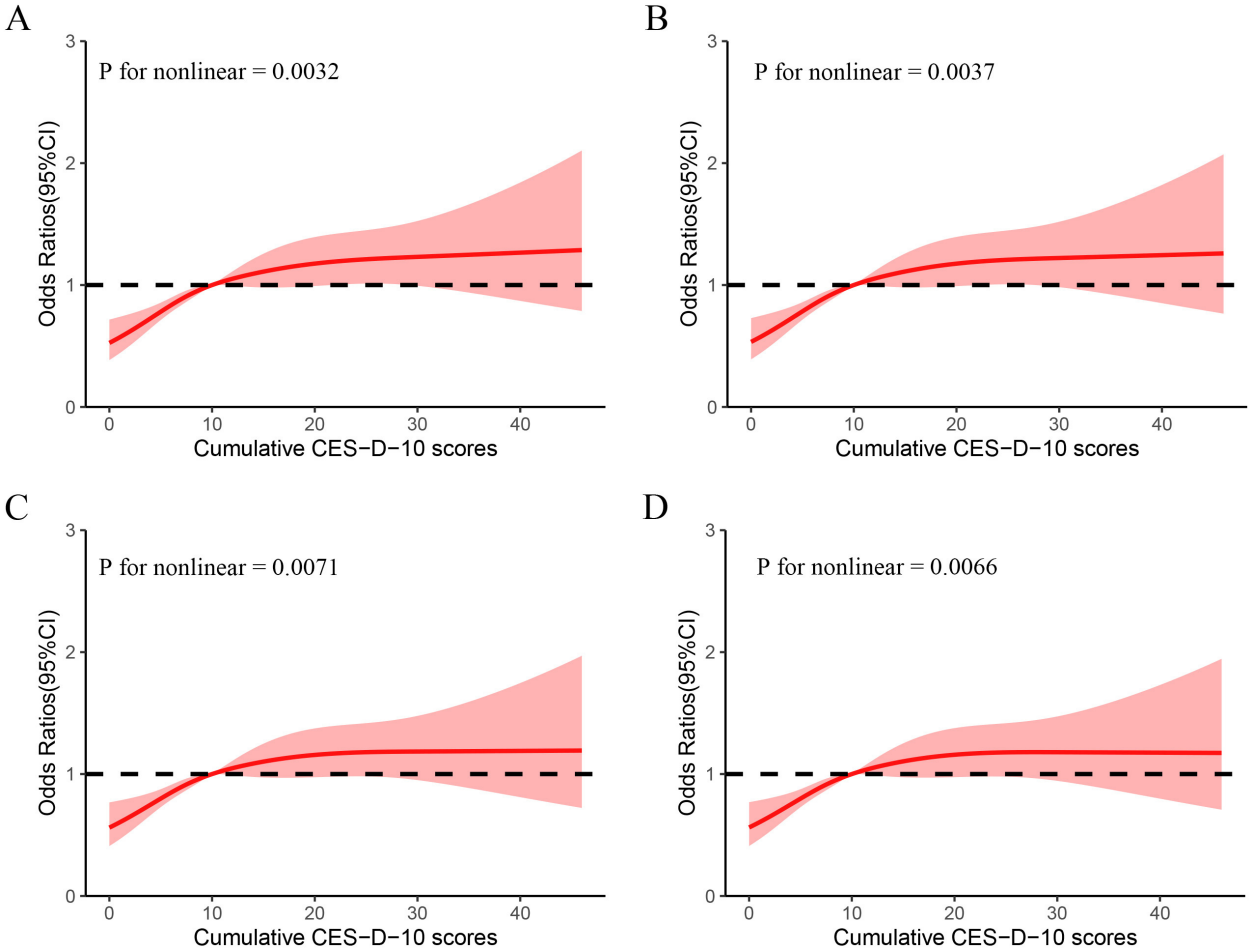
Cubic model of the association between different classes and cumulative CES-D-10 scores. **(A)** Non-adjusted; **(B)** adjusted for age, gender; **(C)** adjusted for age, gender, education, marital status, Hukou, smoking status, and drinking status; **(D)** adjusted for factors in C and history of hypertension, diabetes, heart disease, stroke, dyslipidemia, and lung disease.

New-onset DSI developed in 1,061 participants (32.8%) during the 3 years of follow-up. Multiple logistic regression showed that high levels of CES-D-10 scores were significantly associated with new onset DSI after adjustment for potential confounders, as seen in Model III. Comparing to class 1, the ORs for new-onset DSI were 1.50 (1.25, 1.80), 1.50 (1.15, 1.97), 1.54 (1.24, 1.92), and 1.68 (1.20, 2.35), representing for class 1, class 2, class 3, class 4 and class 5. Divide the cumulative CES-D-10 scores into five groups: Q1 to Q5. This significant relationship remains consistent with previous. In addition, in Model III, using the group with CES-D-10 <10 as a reference, the OR of the group with CES-D-10 >10 was 1.23 (1.02, 1.49). The dose-response curve indicated a positive association between CES-D-10 scores and the risk of new-onset DSI, as shown in Figure 3.

### 3.3 Subclass analyses

Stratified analyses were conducted to further explore the association between CES-D-10 scores and the risk of new-onset DSI in various subgroups in Table 3. Notably, there was a positive association in the subgroup of hukou. However, this association was not observed in other subgroups.

**Table 3:** Subgroup analysis of the associations between different classes and dual sensory impairment.

**Characteristics**	**Case**	**Class 1**	**Class 2**	**Class 3**	**Class 4**	**Class 5**	***P* for interaction**
**Age (years)**							0.145
Q1 (45–51)	1,052	Ref	1.42 (1.02, 1.97) 0.038	1.96 (1.21, 3.18) 0.007	1.43 (0.97, 2.10) 0.073	1.15 (0.59, 2.23) 0.682	
Q2 (52–60)	1,092	Ref	1.63 (1.19, 2.24) 0.003	1.23 (0.74, 2.05) 0.420	1.90 (1.27, 2.84) 0.002	3.45 (1.90, 6.27) <0.001	
Q3 (61–90)	1,093	Ref	1.47 (1.07, 2.01) 0.017	1.42 (0.91, 2.21) 0.119	1.38 (0.96, 1.99) 0.087	1.30 (0.75, 2.27) 0.346	
**Gender**							0.530
Male	1,675	Ref	1.58 (1.23, 2.03) <0.001	1.84 (1.22, 2.78) 0.004	1.66 (1.21, 2.27) 0.002	1.25 (0.64, 2.43) 0.514	
Female	1,562	Ref	1.39 (1.05, 1.83) 0.020	1.30 (0.90, 1.87) 0.162	1.44 (1.05, 1.97) 0.022	1.76 (1.17, 2.64) 0.007	
**Education level**							0.359
Primary school or lower	1,849	Ref	1.37 (1.07, 1.76) 0.011	1.31 (0.94, 1.83) 0.114	1.31 (0.99, 1.74) 0.057	1.53 (1.04, 2.25) 0.033	
Secondary school or higher	1,388	Ref	1.67 (1.26, 2.20) <0.001	1.85 (1.16, 2.94) 0.009	2.05 (1.43, 2.93) <0.001	1.84 (0.89, 3.80) 0.101	
**Current married**							0.745
No	316	Ref	1.62 (0.83, 3.16) 0.159	2.24 (1.02, 4.93) 0.045	1.52 (0.71, 3.27) 0.286	1.41 (0.56, 3.57) 0.463	
Yes	2,921	Ref	1.48 (1.22, 1.79) <0.001	1.42 (1.06, 1.90) 0.020	1.54 (1.22, 1.94) <0.001	1.72 (1.19, 2.49) 0.004	
**Hukou**							0.014
Agriculture	2,433	Ref	1.34 (1.09, 1.65) 0.006	1.46 (1.08, 1.96) 0.013	1.36 (1.06, 1.75) 0.015	1.84 (1.28, 2.64) 0.001	
Others	804	Ref	2.11 (1.44, 3.09) <0.001	1.69 (0.86, 3.33) 0.131	2.45 (1.52, 3.95) <0.001	0.65 (0.21, 2.02) 0.457	
**Smoking status**							0.477
No	2,367	Ref	1.47 (1.19, 1.83) <0.001	1.41 (1.03, 1.94) 0.032	1.51 (1.17, 1.94) 0.002	1.83 (1.26, 2.64) 0.001	
Yes	870	Ref	1.53 (1.08, 2.18) 0.018	1.92 (1.13, 3.26) 0.015	1.66 (1.06, 2.60) 0.027	0.91 (0.36, 2.28) 0.834	
**Drinking status**							0.706
No	1,986	Ref	1.46 (1.15, 1.85) 0.002	1.38 (0.99, 1.91) 0.056	1.50 (1.13, 1.98) 0.005	1.48 (1.00, 2.18) 0.048	
Yes	1,251	Ref	1.55 (1.16, 2.07) 0.003	1.79 (1.10, 2.92) 0.019	1.65 (1.15, 2.38) 0.007	2.60 (1.27, 5.33) 0.009	
**Hypertension**							0.985
No	2,463	Ref	1.49 (1.21, 1.83) <0.001	1.48 (1.08, 2.03) 0.015	1.51 (1.17, 1.93) 0.001	1.64 (1.10, 2.44) 0.016	
Yes	774	Ref	1.59 (1.08, 2.35) 0.019	1.68 (0.99, 2.87) 0.057	1.77 (1.11, 2.83) 0.017	1.86 (0.97, 3.55) 0.061	
**Diabetes**							0.639
No	3,037	Ref	1.53 (1.27, 1.85) <0.001	1.56 (1.18, 2.07) 0.002	1.59 (1.26, 1.99) <0.001	1.71 (1.20, 2.42) 0.003	
Yes	200	Ref	1.12 (0.49, 2.60) 0.787	0.64 (0.20, 2.06) 0.457	0.95 (0.35, 2.57) 0.912	1.33 (0.33, 5.25) 0.689	
**Heart disease**							0.547
No	2,925	Ref	1.54 (1.27, 1.86) <0.001	1.61 (1.21, 2.14) 0.001	1.55 (1.23, 1.95) <0.001	1.55 (1.06, 2.26) 0.023	
Yes	312	Ref	1.11 (0.57, 2.17) 0.760	0.92 (0.39, 2.13) 0.838	1.47 (0.69, 3.13) 0.317	1.90 (0.84, 4.29) 0.122	
**Stroke**							0.188
No	3,175	Ref	1.51 (1.26, 1.82) <0.001	1.51 (1.15, 1.99) 0.003	1.52 (1.22, 1.90) <0.001	1.63 (1.16, 2.31) 0.005	
Yes	62	Ref	0.57 (0.09, 3.50) 0.545	2.73 (0.28, 26.81) 0.390	5.63 (0.47, 67.04) 0.171	4.80 (0.43, 53.23) 0.201	
**Dyslipidemia**							0.558
No	2,847	Ref	1.47 (1.21, 1.79) <0.001	1.58 (1.18, 2.10) 0.002	1.47 (1.17, 1.86) 0.001	1.64 (1.14, 2.35) 0.007	
Yes	390	Ref	1.85 (1.03, 3.31) 0.038	1.25 (0.53, 2.97) 0.608	2.44 (1.24, 4.81) 0.010	1.96 (0.73, 5.25) 0.179	
**Lung disease**							0.475
No	3,003	Ref	1.53 (1.26, 1.85) <0.001	1.46 (1.10, 1.94) 0.009	1.57 (1.25, 1.98) <0.001	1.76 (1.23, 2.52) 0.002	
Yes	234	Ref	1.07 (0.52, 2.20) 0.861	2.33 (0.84, 6.48) 0.104	1.14 (0.50, 2.62) 0.754	1.01 (0.34, 3.00) 0.990	

In addition to the stratification variables themselves, age, gender, education, marital status, hukou, current smoking status, drinking status, hypertension, diabetes, heart disease, stroke, dyslipidemia, and lung disease were adjusted.

Subgroup analysis based on class 1, other subgroups showed a higher risk of DSI in married people, drinking patients, people without tobacco use, people without hypertension, people without diabetes, people without heart disease, people without stroke, people without dyslipidemia, and people without lung disease compared to other subgroups.

### 3.4 Additional analyses

Demographic and baseline characteristics after dividing the cumulative CES-D-10 into five groups (Q1–Q5) are shown in Supplementary Table S1. Results following analysis of the five groups using subgroups are shown in Supplementary Table S2, where we did not find a positive association between CES-D-10 and DSI in the hukou subgroup.

## 4 Discussion

We selected 3,237 participants from the CHARLS database to investigate the association between depression scores and new-onset of DSI. The findings showed the change in CES-D-10 were independently associated with the risk of new-onset DSI in the individuals aged 45 years and older. The risk of DSI increases when cumulative CES-D-10 increases. Additionally, a interaction effect between the CES-D-10 and hukou could be observed, indicating that the relationship between CES-D-10 and the incidence of DSI was most prominent in people who had a rural hukou. In a word, the relationship between cumulative CES-D-10 and new-onset DSI presented a non-linear relationship. Our study suggests that detecting long-term changes in CES-D-10 may be useful for early identification of individuals at high risk for DSI and have positive implications in improving quality of life in older adults.

Carrière et al. found evidence of bidirectional association of VI with depression in a French cohort. Carrière et al. reported an inverse association between baseline depression and incident distance visual function (19). Frank et al. found a significant bidirectional association between self-reported VI and depression symptoms (20). Zhang at al. reported a bidirectional associations between sensorineural hearing loss and depression (21). Liu et al. found that the association of self-reported VL, HL, and DSL with depressive symptoms was bidirectional (22). Obtaining these inconsistent results may be because different instruments have been used to measure vision, hearing, and depression in different studies. For example, Carrière et al. used the 20-item Center for Epidemiologic Studies–Depression scale (CES–D) to assess depressive symptoms. Frank et al. employed the Patient Health Questionnaire for Depression (PHQ-4) to assess depressive symptoms. Liu et al. utilized CESD-10 as a survey tool and used a validated threshold value to determine the presence of depressive symptoms in participant. All three studies considered depression as a dichotomous categorical variable representing the presence or absence of depression. In this study, we not only investigated the relationship between depression and DSI in dichotomous variables, but also deeply explored the relationship between different depression score changes and DSI.

In the logistic regression analysis, each model for the class 1 to the class 5 grouped by cluster showed that the worse the subjects' management of depressive state, the higher the probability of new-onset DSI. Previous studies have shown a significant association between alterations in depressive symptoms and subsequent falls in older adults (37). Changes in CES-D-10 scores can predict the occurrence of fall events, and this study also showed that this score can predict the occurrence of DSI. The increased risk of new-onset DSI among individuals with high CES-D-10 scores shows that mental health is an important component of comprehensive DSI prevention strategies. In addition, DSI leads to social isolation accompanied by depression (12), and functional limitations partially mediate the relationship between DSI and depressive symptoms in Chinese older adults (12). Sensory disturbances and dual sensory disturbances exacerbate the risk of depression (1, 12, 17, 18, 38). Our findings suggest that the worse the subjects' management of depressive state, the higher the probability of new-onset DSI. There were two possible explanations for this phenomenon: (1) Dysfunction of neurotransmitters in depressed patients may have a negative impact on vision such as dysfunction of the inhibitory neurotransmitter GABA may result in adverse effects on the visual system as a whole (39). However, neurotransmitters may play a relatively minor role in explaining visual loss in participants with high depression scores compared to depressive behavioral impacts such as non-compliance with clinical recommendations and treatment and poor rehabilitation (40). (2) Individuals with higher depression scores, particularly older Chinese adults, may not actively seek routine vision and hearing care, eventually causing VI or HI, which in turn leads to the development of DSI. Therefore, we believe that depression score has prognostic value for new-onset DSI in middle-aged and older adult Chinese adults.

In this study, we observed an interaction between CES-D-10 scores and hukou, but this result was not observed in Supplementary Table S2. This may be explained by the following mechanisms: First, hukou and depression may be closely related to social factors, such as loneliness and lack of social support (12). Second, differences in living environment, economic background and medical care levels may play an important role in affecting the relationship between depression levels and new-onset dual sensory disturbances, thereby increasing the risk of DSI. Then, Individuals who live in an urban district may have a rural hukou and vice versa. Hukou status affects many aspects of life in China such as buying a house, buying a car, children's school enrollment and other welfare. Although the group of cumulative CES-D-10 scores has an overall summary of depression over time, there is a lack of accurate identification of trends in depression development in participants over time, so no interaction between CES-D-10 scores and hukou was observed in Supplementary Table S2. However, the pathogenesis of DSI may be very complex and further studies are needed to deeply investigate the details and associated factors involved. In the RCS model we clarify the non-linear association between cumulative CES-D-10 and DSI. This association may be related to the following aspects. The cumulative CES-D-10 scores represents the level of depression and physical condition of the subject through 2013–2015, while the long-term high CES-D-10 scores represents the subject's long-term negative emotionally dominant state, in which people are more sensitive and irritable, and this state may accelerate the visual impairment process in patients with isolated hearing impairment, resulting in the development of DSI, which is an irreversible risk event. Similarly, people with visual impairment may have this mechanism.

According to existing studies, no previous studies have used cluster analysis to classify the change in CES-D-10 scores. In this study, we propose a novel method for classifying the change in CES-D-10 values using cluster analysis. Each category in the analysis corresponded to a distinct subpopulation, where individuals with persistently low CES-D-10 showed the lowest risk, whereas those with highest CES-D-10 and rising trends showed the highest risk. Notably, our study sample included representative cross-sections of healthy individuals from different regions of China. By focusing on dynamic processes, our study provides further evidence to clarify the association between CES-D-10 and DSI. Specifically, our RCS model elucidates the non-linear association between cumulative CES-D-10 scores and DSI. However, the underlying mechanistic explanation for this observed association remains uncertain. To our knowledge, this is the first study to use CES-D-10 scores to predict DSI and has achieved remarkable results, that the occurrence of DSI, an irreversible risk event, can be reduced by improving the long-term psychological status of older adults. Given the relationship between depressive symptoms, the changes in depressive state, and DSI, healthcare providers should regularly evaluate depressive symptoms in older adults, focus on the change in depressive symptoms, and promptly initiate psychotherapeutic measures, which may decrease the probability of new-onset DSI events and reduce costs of care in older adults.

This study has several limitations, first, it included only a 2-year questionnaire and may lack a comprehensive assessment of CES-D-10. Second, this study used an observational design and must acknowledge the existence of selection bias due to loss of follow-up. Third, the study population is middle-aged and older adult people aged 45 years and older in China, and more studies are needed to confirm the situation of other ethnic groups and countries. Finally, due to study limitations, we could not determine the causal relationship between CES-D-10 scores and new-onset DSI, and further experimental studies are needed.

## 5 Conclusion

In summary, in our study, subjects with the worst control of depressive symptoms showed a higher risk of new-onset DSI, which may be mitigated by timely intervention in the psychological status of middle-aged and older adult people. Our study provides us with clues that early depression screening and assessment in Chinese middle-aged and older adults' care services may help identify those at higher risk of developing new onset DSI. And encourage these depressed people to carry out corresponding psychological intervention, so as to greatly improve the quality of life of the middle-aged and older adult population, while reducing the huge burden on the medical industry.

## Data Availability

Publicly available datasets were analyzed in this study. This data can be found here: http://charls.pku.edu.cn.
