# Supplementary material for: Change in depressive symptom scores to assess the risk of new-onset dual sensory impairment in middle-aged and older adults: a nationwide cohort study
Source: Front Public Health. 2025 Mar 26;13:1520552. doi: 10.3389/fpubh.2025.1520552 (PMC11978621; doi:10.3389/fpubh.2025.1520552)
Supplement: Supplementary file 1 [file Table_1.DOCX]

**Table S1** **Characteristics of the study according to** **the quartile of cumulative CES-D-10 scores.**

| **Characteristics** | **Q 1** | **Q 2** | **Q 3** | **Q 4** | **Q 5** | P-value |
| --- | --- | --- | --- | --- | --- | --- |
| n | 535 | 688 | 665 | 685 | 664 |  |
| Age | 56.59 ± 8.48 | 57.54 ± 8.65 | 57.64 ± 8.54 | 57.50 ± 9.10 | 57.80 ± 8.76 | 0.116 |
| Gender(male) | 331 (61.87%) | 401 (58.28%) | 349 (52.48%) | 364 (53.14%) | 230 (34.64%) | <0.001 |
| Education level |  |  |  |  |  | <0.001 |
| Primary school or lower | 237 (44.30%) | 349 (50.73%) | 374 (56.24%) | 409 (59.71%) | 480 (72.29%) |  |
| Secondary school or higher | 298 (55.70%) | 339 (49.27%) | 291 (43.76%) | 276 (40.29%) | 184 (27.71%) |  |
| Current married | 496 (92.71%) | 646 (93.90%) | 606 (91.13%) | 612 (89.34%) | 561 (84.49%) | <0.001 |
| Hukou |  |  |  |  |  | <0.001 |
| Agriculture | 350 (65.42%) | 499 (72.53%) | 496 (74.59%) | 531 (77.52%) | 557 (83.89%) |  |
| Others | 185 (34.58%) | 189 (27.47%) | 169 (25.41%) | 154 (22.48%) | 107 (16.11%) |  |
| Smoking | 156 (29.16%) | 203 (29.51%) | 188 (28.27%) | 172 (25.11%) | 151 (22.74%) | 0.023 |
| Drinking | 257 (48.04%) | 291 (42.30%) | 262 (39.40%) | 259 (37.81%) | 182 (27.41%) | <0.001 |
| Hypertension | 121 (22.62%) | 161 (23.40%) | 158 (23.76%) | 156 (22.77%) | 178 (26.81%) | 0.386 |
| Diabetes | 30 (5.61%) | 35 (5.09%) | 43 (6.47%) | 49 (7.15%) | 43 (6.48%) | 0.552 |
| Heart disease | 42 (7.85%) | 59 (8.58%) | 57 (8.57%) | 58 (8.47%) | 96 (14.46%) | <0.001 |
| Stroke | 4 (0.75%) | 11 (1.60%) | 11 (1.65%) | 13 (1.90%) | 23 (3.46%) | 0.011 |
| Dyslipidemia | 55 (10.28%) | 84 (12.21%) | 80 (12.03%) | 86 (12.55%) | 85 (12.80%) | 0.714 |
| Lung disease | 27 (5.05%) | 45 (6.54%) | 53 (7.97%) | 43 (6.28%) | 66 (9.94%) | 0.011 |
| Depressive symptoms | 0 (0.00%) | 0 (0.00%) | 12 (1.80%) | 137 (20.00%) | 445 (67.02%) | <0.001 |
| CES-D-10_2013_ | 0.87 ± 1.08 | 3.09 ± 1.79 | 4.87 ± 2.26 | 7.23 ± 2.70 | 12.26 ± 5.11 | <0.001 |
| CES-D-10_2015_ | 0.70 ± 1.05 | 2.46 ± 1.79 | 4.57 ± 2.27 | 6.86 ± 2.79 | 13.33 ± 5.22 | <0.001 |
| Cumulative CES-D-10 | 1.58 ± 1.21 | 5.55 ± 1.10 | 9.43 ± 1.07 | 14.09 ± 1.73 | 25.59 ± 6.92 | <0.001 |
| Dual sensory impairment | 124 (23.18%) | 212 (30.81%) | 215 (32.33%) | 262 (38.25%) | 248 (37.35%) | <0.001 |

Continuous variables were expressed as mean±standard deviation (SD) in case of normal distribution and compared between two groups by Kruskal-Wallis rank sum test. If the count variable had a theoretical number <10, Fisher's exact probability test was used. Categorical variables are presented as counts (percentages) and compared by Chi-square test.

**Table S2 Subgroup analysis of the associations between the quartile of cumulative CES-D-10 scores and dual sensory impairment.**

|  | **Case** | **Q 1** | **Q 2** | **Q 3** | **Q 4** | **Q 5** | ***P* for interaction** |
| --- | --- | --- | --- | --- | --- | --- | --- |
| Age(years) |  |  |  |  |  |  | 0.209 |
| Q1(45 - 51) | 1052 | Ref | 1.25 (0.80, 1.94) 0.332 | 1.19 (0.76, 1.87) 0.446 | 1.31 (0.85, 2.03) 0.221 | 1.56 (1.00, 2.45) 0.052 |  |
| Q2(52 - 60) | 1092 | Ref | 1.65 (1.04, 2.63) 0.035 | 1.50 (0.93, 2.42) 0.099 | 2.73 (1.72, 4.34) <0.001 | 2.48 (1.53, 4.03) <0.001 |  |
| Q3(61 - 90) | 1093 | Ref | 1.44 (0.91, 2.27) 0.116 | 1.89 (1.20, 2.96) 0.006 | 2.06 (1.32, 3.24) 0.002 | 1.64 (1.04, 2.58) 0.035 |  |
| Gender |  |  |  |  |  |  | 0.608 |
| male | 1675 | Ref | 1.34 (0.95, 1.88) 0.096 | 1.69 (1.19, 2.38) 0.003 | 2.00 (1.43, 2.81) <0.001 | 1.83 (1.25, 2.69) 0.002 |  |
| female | 1562 | Ref | 1.57 (1.04, 2.35) 0.031 | 1.35 (0.90, 2.02) 0.147 | 1.84 (1.24, 2.74) 0.003 | 1.77 (1.21, 2.60) 0.004 |  |
| Education level |  |  |  |  |  |  | 0.744 |
| Primary school or lower | 1849 | Ref | 1.51 (1.05, 2.19) 0.027 | 1.54 (1.07, 2.22) 0.019 | 1.83 (1.28, 2.61) <0.001 | 1.74 (1.22, 2.48) 0.002 |  |
| Secondary school or higher | 1388 | Ref | 1.34 (0.92, 1.94) 0.123 | 1.44 (0.98, 2.11) 0.062 | 2.13 (1.47, 3.11) <0.001 | 1.92 (1.26, 2.93) 0.003 |  |
| Current married |  |  |  |  |  |  | 0.734 |
| No | 316 | Ref | 1.33 (0.48, 3.71) 0.589 | 2.13 (0.83, 5.45) 0.114 | 1.71 (0.68, 4.28) 0.251 | 1.77 (0.73, 4.28) 0.208 |  |
| Yes | 2921 | Ref | 1.41 (1.08, 1.85) 0.012 | 1.44 (1.09, 1.89) 0.009 | 1.95 (1.49, 2.55) <0.001 | 1.78 (1.35, 2.36) <0.001 |  |
| Hukou |  |  |  |  |  |  | 0.117 |
| Agriculture | 2433 | Ref | 1.25 (0.92, 1.69) 0.151 | 1.38 (1.02, 1.87) 0.036 | 1.64 (1.22, 2.20) 0.001 | 1.70 (1.26, 2.30) <0.001 |  |
| Others | 804 | Ref | 2.18 (1.29, 3.69) 0.004 | 1.93 (1.13, 3.30) 0.017 | 3.36 (1.97, 5.72) <0.001 | 1.98 (1.08, 3.63) 0.027 |  |
| Current smoking status |  |  |  |  |  |  | 0.553 |
| No | 2367 | Ref | 1.58 (1.16, 2.16) 0.004 | 1.70 (1.25, 2.31) <0.001 | 2.07 (1.53, 2.80) <0.001 | 1.90 (1.39, 2.59) <0.001 |  |
| Yes | 870 | Ref | 1.08 (0.67, 1.75) 0.754 | 1.08 (0.66, 1.77) 0.751 | 1.64 (1.01, 2.67) 0.046 | 1.62 (0.97, 2.70) 0.064 |  |
| Drinking status |  |  |  |  |  |  | 0.571 |
| No | 1986 | Ref | 1.69 (1.19, 2.42) 0.004 | 1.69 (1.18, 2.41) 0.004 | 2.10 (1.48, 2.98) <0.001 | 1.88 (1.33, 2.67) <0.001 |  |
| Yes | 1251 | Ref | 1.16 (0.79, 1.72) 0.444 | 1.33 (0.90, 1.98) 0.151 | 1.81 (1.23, 2.66) 0.003 | 1.89 (1.23, 2.89) 0.004 |  |
| Hypertension |  |  |  |  |  |  | 0.126 |
| No | 2463 | Ref | 1.58 (1.17, 2.12) 0.003 | 1.50 (1.11, 2.02) 0.008 | 1.85 (1.38, 2.48) <0.001 | 1.88 (1.39, 2.55) <0.001 |  |
| Yes | 774 | Ref | 0.94 (0.53, 1.65) 0.828 | 1.50 (0.87, 2.59) 0.142 | 2.28 (1.33, 3.91) 0.003 | 1.62 (0.93, 2.79) 0.086 |  |
| Diabetes |  |  |  |  |  |  | 0.646 |
| No | 3037 | Ref | 1.40 (1.07, 1.83) 0.014 | 1.51 (1.15, 1.97) 0.003 | 1.99 (1.53, 2.59) <0.001 | 1.81 (1.38, 2.38) <0.001 |  |
| Yes | 200 | Ref | 1.99 (0.58, 6.80) 0.272 | 1.43 (0.43, 4.70) 0.561 | 1.29 (0.40, 4.19) 0.674 | 1.39 (0.41, 4.70) 0.598 |  |
| Heart disease |  |  |  |  |  |  | 0.260 |
| No | 2925 | Ref | 1.41 (1.07, 1.84) 0.014 | 1.45 (1.11, 1.91) 0.007 | 1.99 (1.53, 2.60) <0.001 | 1.73 (1.31, 2.29) <0.001 |  |
| Yes | 312 | Ref | 1.87 (0.70, 4.98) 0.213 | 2.61 (1.00, 6.83) 0.051 | 1.46 (0.54, 3.94) 0.455 | 2.53 (1.00, 6.38) 0.049 |  |
| Stroke |  |  |  |  |  |  | 0.436 |
| No | 3175 | Ref | 1.40 (1.08, 1.82) 0.011 | 1.49 (1.14, 1.93) 0.003 | 1.91 (1.47, 2.47) <0.001 | 1.76 (1.35, 2.30) <0.001 |  |
| Yes | 62 | Ref | NA | NA | NA | NA |  |
| Dyslipidemia |  |  |  |  |  |  | 0.553 |
| No | 2847 | Ref | 1.42 (1.08, 1.87) 0.011 | 1.43 (1.09, 1.88) 0.011 | 1.91 (1.46, 2.51) <0.001 | 1.74 (1.32, 2.30) <0.001 |  |
| Yes | 390 | Ref | 1.73 (0.70, 4.30) 0.237 | 2.82 (1.16, 6.85) 0.022 | 2.49 (1.03, 6.03) 0.043 | 2.87 (1.16, 7.08) 0.022 |  |
| Lung disease |  |  |  |  |  |  | 0.127 |
| No | 3003 | Ref | 1.46 (1.12, 1.92) 0.006 | 1.54 (1.18, 2.02) 0.002 | 2.07 (1.59, 2.70) <0.001 | 1.78 (1.35, 2.35) <0.001 |  |
| Yes | 234 | Ref | 0.91 (0.32, 2.62) 0.864 | 0.88 (0.32, 2.45) 0.808 | 0.66 (0.23, 1.93) 0.448 | 1.71 (0.64, 4.59) 0.286 |  |

In addition to the stratification variables themselves, age, gender, education, marital status, hukou, current smoking status, drinking status, hypertension, diabetes, heart disease, stroke, dyslipidemia and lung disease were adjusted.
